# Measurement properties of the ICECAP-A capability well-being instrument among dermatological patients

**DOI:** 10.1007/s11136-021-02967-2

**Published:** 2021-08-09

**Authors:** Fanni Rencz, Ariel Z. Mitev, Balázs Jenei, Valentin Brodszky

**Affiliations:** 1 Department of Health Economics, Corvinus University of Budapest, 8 Fővám tér, Budapest, 1093 Hungary; 2 Institute of Marketing, Corvinus University of Budapest, 8 Fővám tér, Budapest, 1093 Hungary; 3 Earnings Statistics Section, Quality of Life Statistics Department, Hungarian Central Statistical Office, 5 Keleti Károly u., Budapest, 1024 Hungary

**Keywords:** Capability, Well-being, Quality of life, ICECAP, DLQI, Skindex-16

## Abstract

**Background:**

Capability well-being captures well-being based on people’s ability to do the things they value in life. So far, no capability well-being measures have been validated in dermatological patients.

**Objectives:**

To validate the adult version of the ICEpop CAPability measure (ICECAP-A) in patients with dermatological conditions. We aimed to test floor and ceiling effects, structural, convergent and known-group validity, and measurement invariance.

**Methods:**

In 2020, an online, cross-sectional survey was carried out in Hungary. Respondents with self-reported physician-diagnosed dermatological conditions completed the ICECAP-A, Satisfaction with Life Scale (SWLS), WHO-5 Well-Being Index and two dermatology-specific measures, Dermatology Life Quality Index (DLQI) and Skindex-16.

**Results:**

618 respondents (mean age 51 years) self-reported a physician-diagnosed dermatological condition, with warts, eczema, onychomycosis, acne and psoriasis being the most common. ICECAP-A performed well with no floor and mild ceiling effects. The violation of local independence assumption was found between the attributes of ‘attachment’ and ‘enjoyment’. ICECAP-A index scores correlated strongly with SWLS and WHO-5 (*r*_s_ = 0.597–0.644) and weakly with DLQI and Skindex-16 (*r*_s_ = − 0.233 to − 0.292). ICECAP-A was able to distinguish between subsets of patients defined by education and income level, marital, employment and health status. Multigroup confirmatory factor analysis indicated measurement invariance across most of these subgroups.

**Conclusions:**

This is the first study to validate a capability well-being measure in patients with dermatological conditions. The ICECAP-A was found to be a valid tool to assess capability well-being in dermatological patients. Future work is recommended to test measurement properties of ICECAP-A in chronic inflammatory skin conditions.

**Supplementary Information:**

The online version contains supplementary material available at 10.1007/s11136-021-02967-2.

## Introduction

Dermatological conditions are estimated to contribute to approximately 2% to the global burden of disease expressed in disability-adjusted life years, with dermatitis, including atopic, contact and seborrheic dermatitis, acne vulgaris, urticaria, psoriasis, viral and fungal skin diseases being responsible for the largest burden [1]. The adverse effect of skin diseases on patients’ health-related quality of life (HRQoL) is well-documented [2, 3]. A variety of disease-specific (e.g. Psoriasis Disability Index, Quality of Life Index for Atopic Dermatitis), skin-specific (e.g. Dermatology Life Quality Index, Skindex instrument family) and generic instruments (e.g. EQ-5D, Short-form 36) are used to assess HRQoL in dermatological patients [4]. In addition to HRQoL impact, many dermatological conditions have potential well-being implications for patients. In most societies, attractive and healthy appearance has a particular importance; thus, visible disorders of the skin, hair and nails may create a considerable psychological and social burden that extends beyond health [5]. For example, patients with chronic skin diseases often report to experience lower autonomy, personal growth, life satisfaction, happiness and purpose in life [6, 7].

HRQoL measures may not be able to capture the well-being burden of living with a dermatological disease. Relatively few studies have so far examined the subjective well-being of dermatological patients [7–10], and none of them have investigated capability well-being. The capability approach, drawing on the work of Nobel Laureate economist Amartya Sen, addresses well-being in terms of people’s capabilities that reflect what people are able to do rather than what they actually do (i.e. functioning) [11]. So far, 14 different capability-based well-being questionnaires have been developed for use in healthcare, such as the ICEpop CAPability Measure (ICECAP), Adult Social Care Outcome Toolkit (ASCOT) and Oxford Capability questionnaire-Mental Health (OxCAP-MH) [12, 13]. Over the past decade, these questionnaires have been gaining increasing interest, especially because they may expand the evaluative space in health economic evaluations by allowing to value non-health attributes [12, 13]. In some countries, such as the UK and the Netherlands, health technology assessment bodies recommend the inclusion of capability outcomes in the assessment of health interventions and programmes where the intended benefits from interventions are associated with non-health-related effects (e.g. social or long-term care) [14, 15].

The ICECAP instruments are among the most frequently used capability well-being measures [13]. Previous studies have validated the adult (ICECAP-A) and elderly (ICECAP-O) versions in several mental illnesses, including depression and drug addiction [16–18]; however, little empirical evidence is available on their measurement properties in the context of physical problems [19–22]. So far, the ICECAP measures or other capability-based well-being measures have not been validated in patients with dermatological conditions.

The objective of this study is to validate the ICECAP-A questionnaire in patients with dermatological conditions. We aim to test floor and ceiling effects, structural, convergent and known-group validity and measurement invariance of the ICECAP-A.

## Methods

### Study population

The study received ethics approval from the Research Ethics Committee of the Medical Research Council in Hungary (Reference No. 3857-4/2019/EKU). In February 2020, an internet-based cross-sectional survey was carried out among the general population aged 18 years or over in Hungary. The survey population was recruited from members of an online panel by a survey company using non-probability quota sampling. The online panel had over 150 thousand members who had voluntarily registered to complete surveys in return for earning survey points that could be later redeemed to various rewards (e.g. gift card, prizes). We aimed for representativeness of the Hungarian general public with respect to age, sex, education, place of living and region. Informed consent was obtained from each respondent at the beginning of the survey. We followed the Strengthening the Reporting of Observational Studies in Epidemiology (STROBE) checklist for observational studies [23].

### Questionnaire

A self-administered questionnaire was developed for the survey that recorded information about the presence of dermatological conditions, sociodemographic characteristics, health status, HRQoL and well-being. General health status was measured in two separate questions. First, we used a 0–100 horizontal VAS, with 0 being the ‘worst imaginable health’ and 100 being the ‘best imaginable health’. This scale is widely used to measure health status and demonstrated a good validity and excellent reliability [24]. Secondly, we asked respondents to rate their overall health as very good, good, fair, bad or very bad.

Respondents were queried about the presence of any dermatological conditions in two steps. In the first step, they were asked to indicate if they had any dermatological condition at the time of the survey. For this question, a predefined list of ten dermatological disease categories (acne, basal cell carcinoma, eczema, herpes zoster, onychomycosis, psoriasis, rosacea, tinea pedis, urticaria and warts) and an ‘Other’ response option with an open-ended text box were provided to the respondents. In the second step, subjects that self-reported any dermatological condition were asked to mark those conditions that were diagnosed by a physician. There was no missing data in this survey, as participants could only proceed to the next question if they had responded to the previous one.

### Outcome measures

#### ICECAP-A

ICECAP-A is a measure of capability well-being consisting of the following five attributes: stability (an ability to feel settled and secure), attachment (an ability to have love, friendship and support), autonomy (an ability to be independent), achievement (an ability to achieve and progress in life) and enjoyment (an ability to experience enjoyment and pleasure) [25]. The Hungarian version of ICECAP-A has earlier been validated in a general population sample [26]. We used a value set based on general population preferences in the UK to compute ICECAP-A index scores [27]. These values are anchored on a zero (no capability on any attribute) to one (full capability on all attributes) scale.

#### Dermatology Life Quality Index (DLQI)

DLQI is a skin-specific HRQoL questionnaire consisting of 10 items [28]. It aims to capture the impact of dermatological conditions on the patients’ life over the last week. Each item has four or five possible response options that are scored from 0 (‘not at all’ or ‘not relevant’) to 3 (‘very much’). The scores of individual items are summed to generate a total DLQI score that ranges between 0 (no impact on HRQoL) and 30 (maximum HRQoL impact).

#### Skindex-16

Similarly to the DLQI, Skindex-16 is also a skin-specific HRQoL measure with a one-week recall period [29]. It has 16 individual items that are scored on a continuous bipolar scale with seven boxes anchored by the words ‘never bothered’ (= 0) and ‘always bothered’ (= 6). Responses to the items of Skindex-16 are categorised into three subscales: symptoms, emotions and functioning. Subscale scores are normalized to a 0–100 scale, where higher scores indicate worse HRQoL.

#### Well-being, life satisfaction and happiness

The 5-item World Health Organization Well-Being Index (WHO-5) was administered to measure subjective well-being over the last two weeks [30, 31]. It asks respondents to rate five positively phrased statements on a 0 (none of the time) to 5 (all of the time) scale, so that the final score ranges between 0 and 25. However, this is conventionally transformed to a 0–100 scale, where higher values indicate greater level of well-being. The Satisfaction with Life Scale (SWLS) was used to assess cognitive judgements of one’s life satisfaction [32]. Respondents were asked to indicate their degree of agreement on five items using a seven-point agreement scale with responses ranging from 1 (strongly disagree) to 7 (strongly agree). Total scores for this scale range from 5 to 35 with higher scores suggesting a higher life satisfaction. Furthermore, respondents rated their level of satisfaction with life (SWL) on an 11-point numeric rating scale with endpoints of ‘not satisfied at all’ (= 0) and ‘completely satisfied’ (= 10). A similar numeric scale was used to assess happiness with endpoints of ‘completely unhappy’ (= 0) and ‘completely happy’ (= 10).

### Statistical analyses

Most of our analyses concerned with measurement properties that are relevant in the context of a measure to be used in economic evaluation [33]. These included floor and ceiling effects, structural, convergent and known-group validity and measurement invariance. Most of these measurement properties have been tested in previous ICECAP-A and ICECAP-O validation studies in general population and patient samples [16, 17].

#### Floor and ceiling effects

Descriptive statistics were used to provide an overview of the study population. Floor and ceiling effects were considered present if more than 15% of the respondents scored the worst and best capability level for attributes, or zero or one on ICECAP-A index, respectively [34].

#### Structural validity

Confirmatory factor analysis was carried out to confirm the factor structure of ICECAP-A. Model fit was tested using multiple criteria: *χ*^2^-statistic, comparative fit index (CFI), root-mean-square error of approximation (RMSEA) and Tucker-Lewis index (TLI) with values of a *p* < 0.05 for the *χ*^2^-statistic, CFI ≥ 0.90, RMSEA ≤ 0.08, CFI and TLI ≥ 0.95 as an indication of good fit [35].

#### Convergent validity

We tested convergent validity for each ICECAP-A attribute as well as the index scores by using Spearman’s rank-order correlations. Correlation coefficients (*r*_s_) were considered very weak if < 0.20, weak if 0.20–0.39, moderate if 0.40–0.59 and strong if ≥ 0.60 [36]. We expected strong correlations with life satisfaction measures (SWLS and SWL) [37], moderate correlations with health status VAS [13, 20, 22, 37] and subjective well-being as assessed by the WHO-5 [26] and happiness [38], and weak correlations with skin-specific HRQoL measures (DLQI and Skindex-16) [22].

#### Known-group validity

Known-group validity was assessed by examining the extent to which the ICECAP-A was able to distinguish between groups of respondents differing in a characteristic that was likely to be associated with capability well-being. We hypothesized no associations between capability scores and age or sex, and positive associations of capabilities with better self-perceived health status, being more educated, being married or living in a domestic partnership, being employed and having a higher income level [22, 26, 37, 38]. The differences in median ICECAP-A scores across groups were tested by Mann–Whitney *U* or Kruskal Wallis *H* test.

#### Measurement invariance

Measurement invariance of the ICECAP-A across different subgroups [sex, age (< 65 years vs. ≥ 65 years), level of education, marital status, income and DLQI score (DLQI ≤ 10 vs. DLQI > 10)] was evaluated using multigroup confirmatory factor analysis [39]. DLQI score was split at ten points as DLQI > 10 is considered a ‘very large impact’ of the dermatological condition on patients’ lives [40]. A sequence of configural (i.e. same pattern of factors), metric (i.e. same pattern of factors and loadings) and scalar (i.e. same pattern of factors, loadings and item thresholds) models were tested for each variable. We first examined the fit of the configural model. For the assessment of model fit, *χ*^2^-statistic, TLI, CFI and RMSEA were used. We compared the fit of the unconstrained configural model to the metric model, and then, the metric model to the most constrained scalar model. A decrease in CFI ≤ 0.01 was considered as an evidence for invariance [41]. All the statistical tests were two-sided, and *p* < 0.05 was considered statistically significant. We used SPSS 25.0 and Amos 25.0 (IBM Corp. Armonk, NY) for the data analysis.

## Results

### Characteristics of the study population

**Fig. 1:**
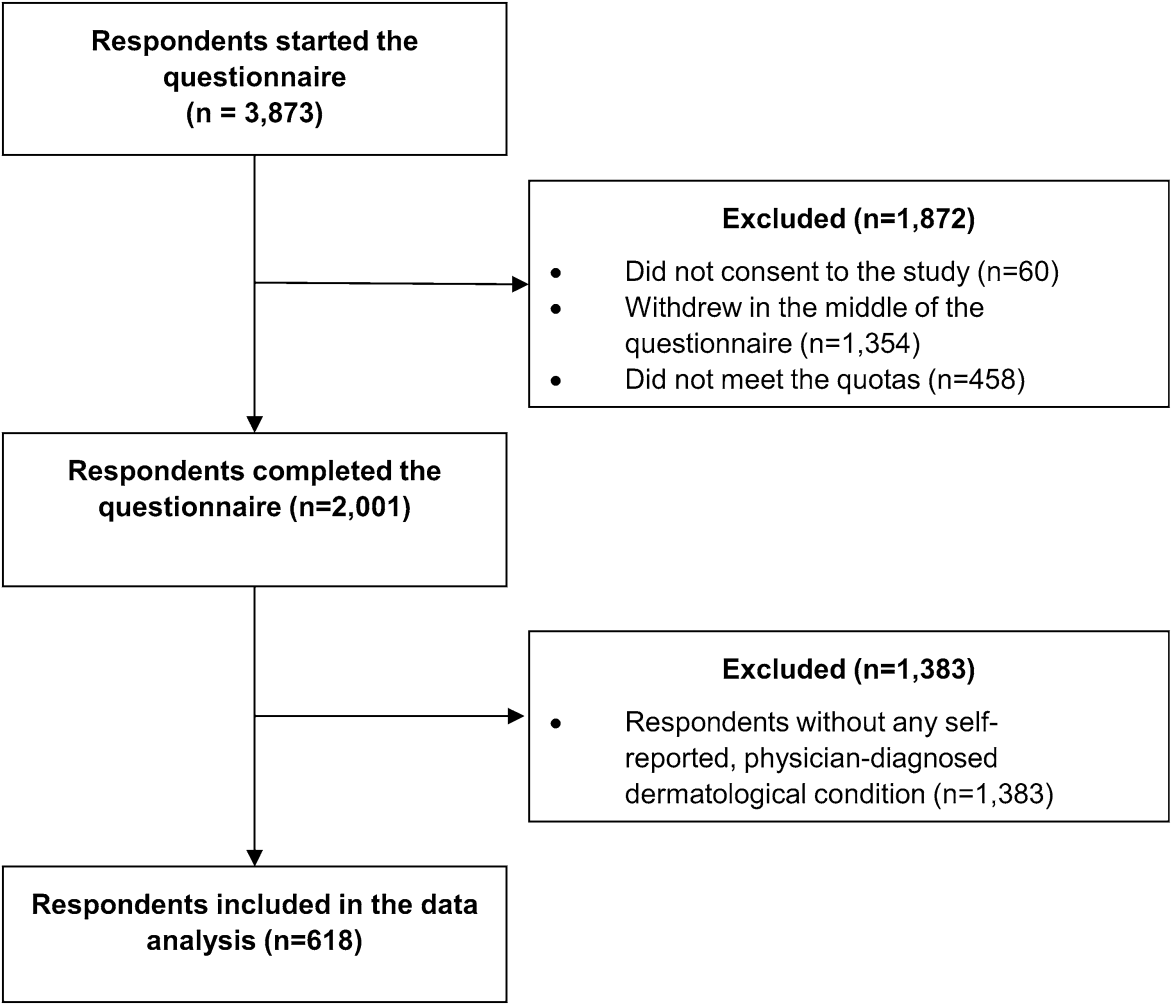
Study flowchart

A total of 3873 people opened the questionnaire, 60 individuals did not consent to the study, 1354 did not finish it and 458 did not meet the quotas, resulting in a final sample of 2001 respondents (Fig. 1). Of the 2001 respondents, 618 individuals self-reported a dermatological condition diagnosed by a physician, and thus, formed the analytical sample for this study. The majority of the sample was female (57.9%), and the mean age was 50.5 ± 16.9 years (Table 1). The most common dermatological conditions in the sample were warts (23.1%), eczema (22.7%), onychomycosis (18.3%), acne (13.4%), psoriasis (13.3%), tinea pedis (7.4%), basal cell carcinoma (5.0%), rosacea (5.0%), urticaria (3.6%), herpes zoster (1.6%) and other (16.5%) (the presence of multiple diseases in one individual was possible). Responses for the open-ended ‘other dermatological condition’ category are presented in Online resource 1.

**Table 1:** Characteristics of the study population and descriptive statistics of ICECAP-A index scores

Variables	*n* (%)	ICECAP-A index score		
		Mean (SD)	Median (IQR)	*p*-value ^*a*^
Total sample	618 (100%)	0.69 (0.20)	0.72 (0.26)	–
Sex				
Female	358 (57.9%)	0.70 (0.20)	0.76 (0.25)	0.191
Male	260 (42.1%)	0.68 (0.20)	0.70 (0.27)	
Age groups (years)				
18–24	42 (6.8%)	0.73 (0.16)	0.76 (0.23)	0.166
25–34	92 (14.9%)	0.71 (0.18)	0.70 (0.26)	
35–44	106 (17.2%)	0.65 (0.23)	0.70 (0.34)	
45–54	106 (17.2%)	0.70 (0.18)	0.75 (0.25)	
55–64	89 (14.4%)	0.65 (0.23)	0.69 (0.34)	
65–74	159 (25.7%)	0.72 (0.17)	0.76 (0.27)	
75 +	24 (3.9%)	0.72 (0.22)	0.77 (0.26)	
Highest level of education				
Primary	31 (5%)	0.61 (0.20)	0.66 (0.33)	< 0.001
Secondary	462 (74.8%)	0.68 (0.20)	0.69 (0.30)	
Tertiary	125 (20.2%)	0.77 (0.16)	0.83 (0.12)	
Marital status				
Married	291 (47.1%)	0.72 (0.19)	0.76 (0.27)	0.019
Divorced	63 (10.2%)	0.63 (0.23)	0.64 (0.41)	
Widowed	40 (6.5%)	0.68 (0.24)	0.78 (0.35)	
Domestic partnership	130 (21%)	0.70 (0.18)	0.71 (0.24)	
Other	94 (15.2%)	0.66 (0.20)	0.69 (0.32)	
Employment				
Full-time	249 (40.3%)	0.72 (0.18)	0.76 (0.27)	< 0.001
Part-time	30 (4.9%)	0.66 (0.18)	0.68 (0.25)	
Retired	190 (30.7%)	0.72 (0.19)	0.76 (0.27)	
Disability pensioner	45 (7.3%)	0.65 (0.20)	0.69 (0.25)	
Student	33 (5.3%)	0.71 (0.17)	0.76 (0.22)	
Unemployed	31 (5%)	0.50 (0.25)	0.53 (0.36)	
Homemaker/housewife	23 (3.7%)	0.62 (0.23)	0.64 (0.35)	
Other	17 (2.8%)	0.66 (0.20)	0.70 (0.41)	
Net monthly household income per capita				
HUF 0–100,623	111 (18%)	0.62 (0.23)	0.67 (0.36)	< 0.001
HUF 100,624–137,500	121 (19.6%)	0.67 (0.21)	0.69 (0.30)	
HUF 137,501–194,454	133 (21.5%)	0.68 (0.18)	0.69 (0.24)	
HUF 194,455–265,165	78 (12.6%)	0.76 (0.17)	0.85 (0.26)	
HUF 265,166 +	91 (14.7%)	0.78 (0.16)	0.82 (0.21)	
Don’t know/refused to answer	84 (13.6%)	0.69 (0.19)	0.70 (0.27)	
Self-perceived health status				
Very good	33 (5.3%)	0.83 (0.14)	0.85 (0.21)	< 0.001
Good	198 (32%)	0.79 (0.14)	0.85 (0.19)	
Fair	264 (42.7%)	0.68 (0.19)	0.69 (0.26)	
Bad	107 (17.3%)	0.56 (0.19)	0.55 (0.28)	
Very bad	16 (2.6%)	0.41 (0.22)	0.44 (0.34)	
Health-related quality of life (Dermatology Life Quality Index)				
DLQI ≤ 10	552 (89.3%)	0.70 (0.19)	0.75 (0.25)	0.002
DLQI > 10	66 (10.7%)	0.61 (0.23)	0.62 (0.37)	

*IQR* interquartile range, *SD* standard deviation

^a^Mann–Whitney *U* test or Kruskal Wallis *H* test

### Health status, HRQoL and well-being

**Fig. 2:**
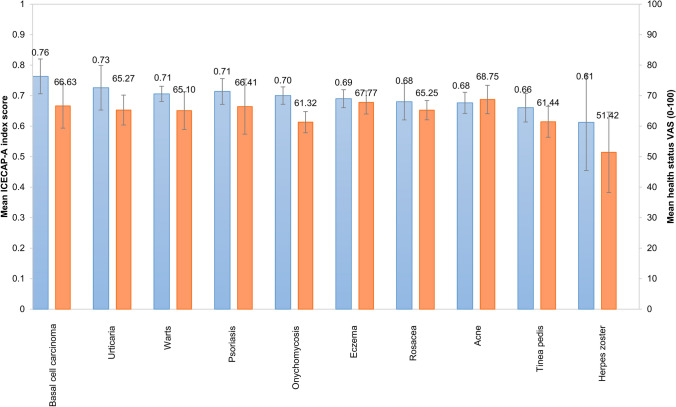
Mean ICECAP-A index and health status VAS scores. *ICECAP-A* ICEpop CAPability measure for adults, *VAS* visual analogue scale

Patients assessed their general health status to a mean of 66.54 ± 23.35 using a 0–100 VAS, ranging from 51.42 in herpes zoster to 68.75 in acne (Fig. 2). The proportion of patients with ‘very good’ or ‘good’ self-reported health status was 37.5%, while 42.7% indicated ‘fair’ and 19.9% ‘bad’ or ‘very bad’. The mean DLQI score in the sample was 3.76 ± 5.03 (Table 2). More problems were reported on the emotions subscale of Skindex-16 (35.92 ± 30.38) compared with symptoms (29.98 ± 28.62) and functioning (22.15 ± 28.31). The mean SWLS, SWL, WHO-5 and happiness scores were 20.08 ± 6.75, 5.93 ± 2.39, 49.69 ± 19.94 and 6.11 ± 2.45, respectively.

**Table 2:** Descriptive statistics of the outcome measures

	Mean	SD	Median	Q1–Q3	Min	Max
ICECAP-A index score (0–1)	0.69	0.20	0.72	0.59–0.85	0.00	1.00
Health status VAS (0–100)	66.54	23.35	71.00	50.00–85.00	0.00	100.00
DLQI (0–30)	3.76	5.03	2.00	0.00–5.56	0.00	29.00
Skindex-16 symptoms (0–100)	29.98	28.62	25.00	4.17–50.00	0.00	100.00
Skindex-16 emotions (0–100)	35.92	30.38	30.95	9.52–57.14	0.00	100.00
Skindex-16 functioning (0–100)	22.15	28.31	6.67	0.00–40.00	0.00	100.00
WHO-5 (0–100)	49.69	19.94	52.00	36.00–64.00	0.00	100.00
SWLS (5–35)	20.08	6.75	20.00	15.00–25.00	5.00	35.00
SWL (0–10)	5.93	2.39	6.00	5.00–8.00	0.00	10.00
Happiness (0–10)	6.11	2.45	7.00	5.00–8.00	0.00	10.00

For DLQI and Skindex, higher scores represent worse outcomes, for all other measures higher scores indicate better outcomes

*DLQI* Dermatology Life Quality Index, *ICECAP-A* ICEpop CAPability measure for adults, *SWLS* Satisfaction with Life Scale, *SWL* Satisfaction with Life visual analogue scale, *VAS* visual analogue scale, *WHO-5* 5-item World Health Organisation Well-Being Index

### Capability well-being outcomes

**Fig. 3:**
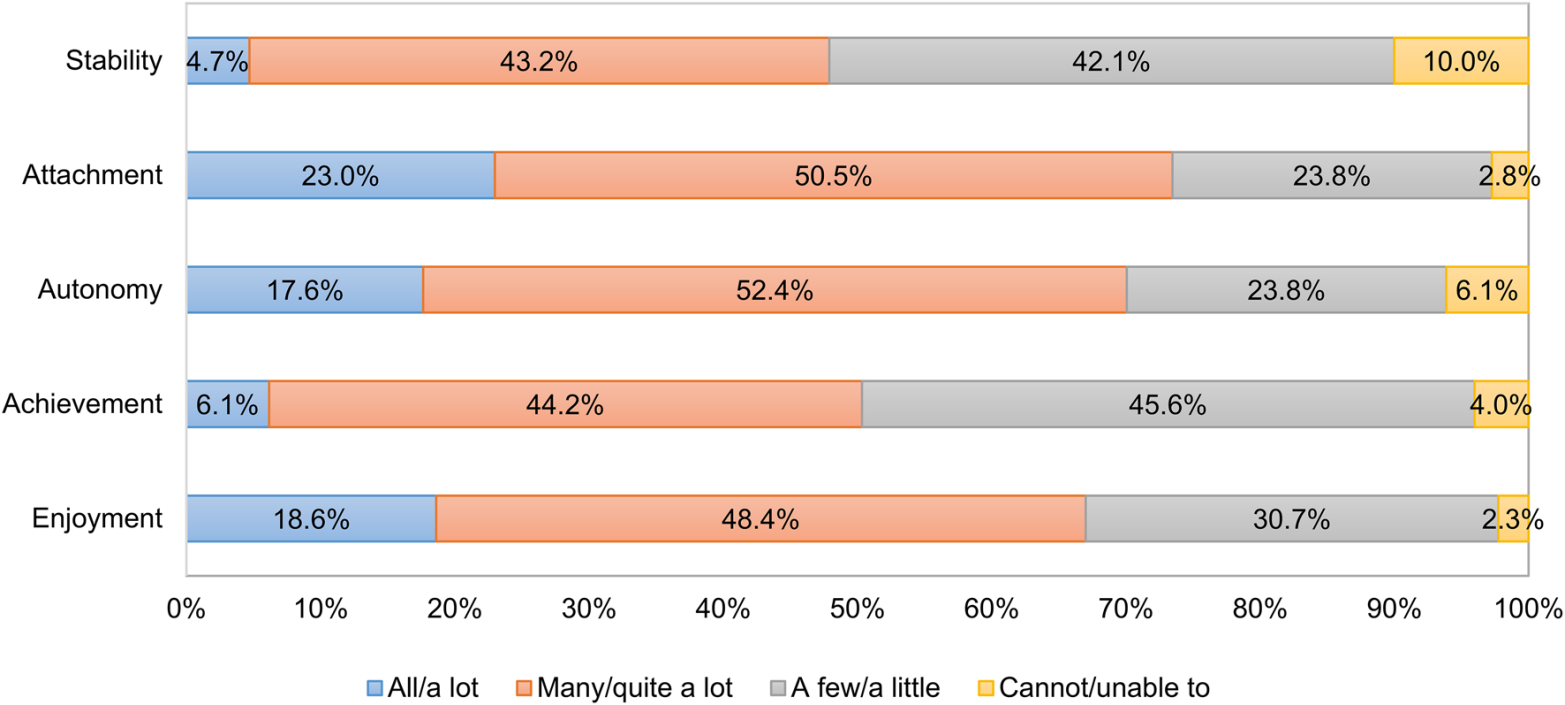
Distribution of responses on the five attributes of ICECAP-A. *ICECAP-A* ICEpop CAPability measure for adults. Percentages may not add up 100% due to reounding

Approximately half of patients recorded responses on the two lowest levels of capability for stability (52.1%) and achievement (49.7%) of ICECAP-A (Fig. 3). The largest proportion of responses in the lowest level was observed for stability (‘I am unable to feel settled and secure in any areas of my life’, 10.0%). Over two-thirds of patients reported no or mild limitations (highest two levels on ICECAP-A) in their capabilities for the attachment (i.e. love friendship, support), autonomy (i.e. being independent) and enjoyment (i.e. enjoyment and pleasure) attributes. The mean ICECAP-A index score was 0.69 ± 0.20, ranging from 0.61 in herpes zoster to 0.76 in basal cell carcinoma (Fig. 2). The impact of various dermatological conditions on health and capability differed; certain conditions (e.g. herpes zoster) had a large effect on both health and capabilities, while others (e.g. acne, eczema) mostly affected capabilities. Mean ICECAP-A index scores of patients with a DLQI ≤ 10 and DLQI > 10 were 0.70 ± 0.19 and 0.61 ± 0.23, respectively (*p* = 0.002).

### Measurement properties of ICECAP-A

#### Floor and ceiling effects

No floor effects were detected for any of the five attributes. Ceiling effects were apparent for the attributes of attachment (23.0%), autonomy (17.6%) and enjoyment (18.6%) (Fig. 3). Five (0.8%) patients reported full capability and four (0.7%) patients were in ‘no capability’; thus, there were no ceiling or floor effects for the index scores.

#### Structural validity

**Fig. 4:**
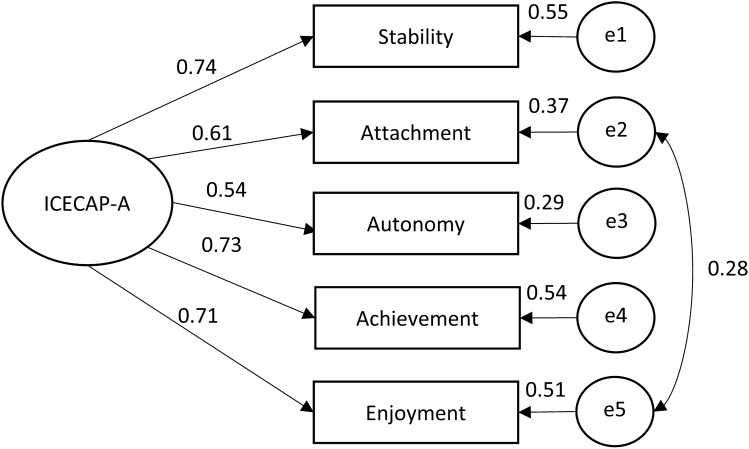
Confirmatory factor analysis of the structure of ICECAP-A. *ICECAP-A* ICEpop CAPability measure for adults

A one-factor model was established in confirmatory factor analysis with the following goodness-of-fit indices: *χ*^2^ = 35.55 (*p* < 0.001), RMSEA = 0.100, TLI = 0.935 and CFI = 0.968. A covariance between the error terms (i.e. local dependency) was identified between the attributes of attachment and enjoyment that improved the model fit [*χ*^2^ = 10.18 (*p* = 0.037), RMSEA = 0.050, TLI = 0.984 and CFI = 0.993] (Fig. 4).

#### Convergent validity

Most hypotheses regarding convergent validity were met. The ICECAP-A index showed a strong correlation with SWLS, SWL, WHO-5 and happiness scores (*r*_s_ = 0.597–0.689) (Table 3). A moderate or strong correlation was found between these four outcomes and all ICECAP-A attributes with the exception of autonomy (*r*_s_ = 0.281–0.607). General health status VAS exhibited a moderate correlation with the ICECAP-A index and weak correlation with the five attributes (*r*_s_ = 0.233–0.449). DLQI and the three Skindex-16 subscales were weakly or very weakly correlated with all five ICECAP-A attributes and index score (*r*_s_ = − 0.123 to − 0.292).

**Table 3:** Convergent validity of ICECAP-A attributes and index scores (Spearman’s correlations)

Outcome measures	ICECAP-A					
	Stability	Attachment	Autonomy	Achievement	Enjoyment	Index score
Health status VAS (0–100)	0.380	0.334	0.233	0.367	0.339	0.449
DLQI (0–30)	− 0.236	− 0.200	− 0.201	− 0.182	− 0.220	− 0.271
Skindex-16 symptoms (0–100)	− 0.215	− 0.184	− 0.144	− 0.123	− 0.194	− 0.233
Skindex-16 emotions (0–100)	− 0.221	− 0.206	− 0.146	− 0.148	− 0.203	− 0.247
Skindex-16 functioning (0–100)	− 0.242	− 0.244	− 0.167	− 0.187	− 0.259	− 0.292
WHO-5 (0–100)	0.559	0.417	0.351	0.518	0.537	0.644
SWLS (5–35)	0.565	0.449	0.281	0.451	0.453	0.597
SWL (0–10)	0.607	0.533	0.339	0.524	0.538	0.689
Happiness (0–10)	0.560	0.574	0.305	0.490	0.586	0.685

*p* < 0.05 for all correlation coefficients

For DLQI and Skindex, higher scores represent worse outcomes, for all other measures higher scores indicate better outcomes

*DLQI* Dermatology Life Quality Index, *ICECAP-A* ICEpop CAPability measure for adults, *SWLS* Satisfaction with Life Scale, *SWL* Satisfaction with Life visual analogue scale, *VAS* visual analogue scale, *WHO-5* 5-item World Health Organisation Well-Being Index

#### Known-group validity

All our hypotheses with respect to validity between known groups of patients were confirmed. Patients with worse self-perceived health status, lower level of education, those not being married or living in domestic partnership, unemployed or with lower income were associated with significantly lower levels of capability-well-being (Table 1). As expected, there were no significant associations between age or sex and ICECAP-A index scores.

#### Measurement invariance

Configural and metric measurement invariance were supported (ΔCFI ≤ 0.01) across all subgroups of patients defined by sex, age, level of education, being married/living in a domestic partnership, income, self-perceived health status and HRQoL as assessed by the DLQI (Table 4). The scalar invariance model demonstrated a slight deterioration in model fit, but only for age, marital status and DLQI groups.

**Table 4:** Measurement invariance (multigroup CFA)

Group	Model	df	*χ*^2^	*p*-value	TLI	RMSEA	CFI	ΔCFI
Sex	Configural	8	13.178	0.106	0.986	0.032	0.995	–
	Metric	12	16.824	0.156	0.991	0.026	0.995	0.000
	Scalar	17	20.736	0.238	0.995	0.019	0.996	0.001
Age	Configural	8	16.227	0.039	0.978	0.041	0.991	–
	Metric	12	17.717	0.125	0.990	0.028	0.994	0.003
	Scalar	17	43.217	< 0.001	0.967	0.050	0.972	**0.022**
Education	Configural	28	75.075	< 0.001	0.944	0.052	0.948	–
	Metric	32	83.280	< 0.001	0.947	0.051	0.943	0.005
	Scalar	37	86.521	< 0.001	0.955	0.047	0.945	0.002
Marital status	Configural	8	11.943	0.154	0.989	0.028	0.996	–
	Metric	12	14.228	0.286	0.996	0.017	0.998	0.002
	Scalar	17	48.516	< 0.001	0.960	0.055	0.966	**0.032**
Income	Configural	68	118.299	< 0.001	0.950	0.037	0.932	–
	Metric	72	119.750	< 0.001	0.955	0.035	0.935	0.003
	Scalar	77	125.028	< 0.001	0.958	0.034	0.935	0.000
Self-perceived health status	Configural	28	52.423	0.003	0.972	0.038	0.974	–
	Metric	32	55.283	0.006	0.976	0.034	0.975	0.001
	Scalar	37	59.238	0.012	0.981	0.031	0.976	0.001
DLQI	Configural	8	17.711	0.024	0.974	0.044	0.990	–
	Metric	12	24.706	0.016	0.977	0.041	0.986	0.004
	Scalar	17	43.692	< 0.001	0.967	0.050	0.972	**0.014**

Groups: sex: female vs. male; age: < 65 years vs. ≥ 65 years; marital status: married/living in a domestic partnership vs. other; income: quintile groups; education: primary/secondary vs. tertiary; self-perceived health status: very good/good vs. fair/bad/very bad; DLQI groups: DLQI ≤ 10 vs. DLQI > 10

Bolded values indicate the lack of measurement invariance

*df* degrees of freedom, *CFA* confirmatory factor analysis, *CFI* comparative fit index, *RMSEA* root-mean-square error of approximation, *TLI* Tucker–Lewis Index

## Discussion

In prior studies, patients with chronic skin diseases, such as psoriasis, pemphigus and morphea, were more likely to be associated with decreased subjective well-being, happiness and life satisfaction [7–9, 42–44]. Yet this is the first study to validate a capability well-being instrument in dermatological patients. Corroborating with previous research on the validity of ICECAP-A in other clinical and population-based studies [16], our findings provide mostly favourable evidence on the psychometric properties of ICECAP-A in a dermatological patient population, including no floor effect, good convergent and known-group validity and established metric and configural invariance across subgroups of patients. However, a mild ceiling effect was present for three attributes, and a local dependence was identified between two of the five attributes.

The sample used for this study was large and heterogeneous representing the most common dermatological conditions in the population, such as warts, eczema, onychomycosis, acne, psoriasis and tinea pedis, among others. There are no data available on the precise prevalence of most dermatological conditions in Hungary. Few existing prevalence estimates from Hungary or the Central and Eastern European region include adult psoriasis (Central Europe: range 0.62–5.32%) and atopic eczema (5%) [45, 46]. In our study, the number of patients with psoriasis and eczema (a wider category than atopic eczema) in the total sample (*n* = 2001) was 82 (4.1%) and 141 (7.0%), respectively, suggesting a good overall representativeness.

Approximately half of the sample reported severe limitations in their stability (feeling settled and secure) and achievement and progress. Mean ICECAP-A index (0.69) was found to be considerably lower than previously reported in other clinical groups (e.g. spinal cord injury 0.76 [37], arthritis 0.81 [47], asthma 0.84 [47], lower urinary tract symptoms 0.85 [22], knee pain 0.89 [21]); but somewhat higher than in patients with opiate dependence (0.66) [48] or depression (0.64) [18]. Moreover, < 1% experienced full capability with regard to all five attributes of ICECAP-A that was 3% and 12% in patients with spinal cord injury and lower urinary tract symptoms, respectively [22, 37]. However, comparison of these scores might be limited by the different language versions of ICECAP-A used in the studies and possible cross-cultural and condition-specific differences in the interpretation of the attributes.

Attributes of ICECAP-A were developed to capture five independent and distinct concepts, three of which, ‘attachment’, ‘autonomy’ and ‘enjoyment’ were aimed to be close equivalents to ‘emotions’, ‘control’ and ‘play’ from Nussbaum’s list of central human capabilities [25]. Notwithstanding, we found the violation of local independence between the attributes of attachment (an ability to have love, friendship and support) and enjoyment (ability to experience enjoyment and pleasure) suggesting an overlap in the content of the attributes. This is not surprising as during the development of the ICECAP-A, the attribute of attachment was reported to be strongly related to the interactions with other people, including partner, close family and good friends, and being around other people may also be a major source of enjoyment and pleasure in life [25, 38].

The ICECAP-A was able to differentiate between 6 of 8 predefined known groups of patients. Higher education and income level, being married or living in a domestic partnership, and better self-perceived general health status or skin-specific HRQoL were associated with higher capability levels, while unemployed patients scored lower on ICECAP-A. The positive associations between higher ICECAP-A scores and marital status, labour force participation and better general health status have earlier been confirmed in patients with type 2 diabetes and spinal cord injury [19, 37]. Evidence is less conclusive with regard to the association of age and ICECAP-A scores. Three earlier studies among members of the general population and female patients with urinary incontinence reported the lack of association between age and ICECAP-A scores [22, 25, 26], whereas another study identified a clear trend towards lower ICECAP-A scores with older age in patients with type 2 diabetes [19].

The measurement equivalence found in this study highlights that ICECAP-A scores can be reliably compared across most known groups of patients. However, scalar equivalence was not confirmed for all subgroups suggesting that certain groups (e.g. being married/living in a domestic partnership or not, DLQI ≤ 10 and DLQI > 10) tend to interpret the attributes of the ICECAP-A in a different way, and differences in scores between these groups are suggested to be treated with caution.

The weak correlation of the ICECAP-A with DLQI and Skindex-16 confirmed that capability wellbeing is a different, but complement construct to HRQoL. It has been increasingly argued to look at outcomes other than health ones, including subjective well-being and capabilities [11, 49, 50]. In addition to health gains, health interventions may offer capability gains too that can represent additional treatment benefits. Health economists and policymakers in healthcare may also see this compelling as adopting the capability wellbeing perspective has already demonstrated to result in different cost-effectiveness estimates, and thus, treatment recommendations for certain health interventions [51, 52]. The National Institute for Health and Care Excellence (NICE) in the UK has already recommended the ICECAP-A and its elderly version, the ICECAP-O questionnaires in its reference case for evaluating social care interventions [15].

Strengths of this study are the large and heterogeneous patient sample and the survey design that ensured a broad representation of the general population. A further strength is the use of validated skin-specific HRQoL measures, such as the DLQI and Skindex-16. To our knowledge, we are the first to test measurement invariance for the ICECAP-A. There are some limitations that are worth noting. First, disadvantages of the online data collection, such as excluding people with no internet access should be considered. In Hungary among the population 16 years or older, the average internet penetration rate at the time of this survey was around 80% [53]. Thus, selection bias might have occurred, to some extent. Secondly, the study was based on self-reported information on diagnosis provided by patients that may be more prone to errors compared to data collection in clinical settings, whereby diagnosis is confirmed by physicians. Thirdly, the survey reached mostly less severe cases as 89.3% had a DLQI score of ≤ 10. Furthermore, we did not have any information on the treatment history of these patients. Several earlier studies from Hungary confirmed that successful treatment and management of skin diseases improve health-related quality of life and well-being of patients [9, 54–59]. Fourthly, in absence of a Hungarian value set for the ICECAP-A, our analyses relied on the ICECAP-A value set for the UK and not that of the Hungarian population, whose values may differ across attributes and levels. Finally, this study had a cross-sectional design that prevented the assessment of other measurement properties, such as test–retest reliability and responsiveness.

In conclusion, the ICECAP-A was found to be a valid tool to measure capability well-being in a dermatological patient population. However, a local dependency was found between the attributes of ‘attachment’ and ‘enjoyment’ that warrants further investigation. Future studies are recommended to assess capability well-being and confirm measurement properties of the ICECAP-A in common chronic inflammatory skin diseases, such as psoriasis, atopic dermatitis and acne. Further research steps also include the validation of the elderly version of ICECAP, the ICECAP-O in dermatological patients as well as the validation of alternative capability measures in this patient population.

## Supplementary Information

Below is the link to the electronic supplementary material.

Supplementary file1 (PDF 297 kb)

## Data Availability

All data of this study are available from the corresponding author upon reasonable request.
