# Supplementary material for: Measurement properties of the ICECAP-A capability well-being instrument among dermatological patients
Source: Qual Life Res. 2021 Aug 9;31(3):903–15. doi: 10.1007/s11136-021-02967-2 (PMC8921030; doi:10.1007/s11136-021-02967-2)
Supplement: Supplementary file 1 — Supplementary file1 (PDF 297 kb) [file 11136_2021_2967_MOESM1_ESM.pdf]

**Electronic Supplementary Material**

Title: Measurement properties of the ICECAP-A capability well-being instrument among dermatological patients
Authors: Fanni Rencz, Ariel Z. Mitev, Balázs Jenei and Valentin Brodszky
Journal: Quality of Life Research

## Supplementary material 1 Other skin conditions reported in the open-ended textbox

| Skin condition* | n | % |
|---|---|---|
| seborrheic dermatitis | 10 | 1.6% |
| naevi | 7 | 1.1% |
| scalp or hair diseases | 7 | 1.1% |
| vitiligo | 7 | 1.1% |
| xeroderma | 6 | 1.0% |
| melanoma | 6 | 1.0% |
| other fungal disease** | 5 | 0.8% |
| polymorphic light eruption | 5 | 0.8% |
| fibroma | 4 | 0.6% |
| herpes | 3 | 0.5% |
| leg ulcer | 3 | 0.5% |
| milia | 3 | 0.5% |
| keratoma | 2 | 0.3% |
| keratosis (unspecified) | 2 | 0.3% |
| lipoma | 2 | 0.3% |
| lymphedema | 2 | 0.3% |
| acroangiodermatitis | 1 | 0.2% |
| actinic keratosis | 1 | 0.2% |
| aphthous stomatitis | 1 | 0.2% |
| capillary damages | 1 | 0.2% |
| clavus | 1 | 0.2% |
| demodex mite | 1 | 0.2% |
| erysipelas | 1 | 0.2% |
| erythroplasia of Queyrat | 1 | 0.2% |
| folliculitis | 1 | 0.2% |
| hidradenitis suppurativa | 1 | 0.2% |
| human papillomavirus infection | 1 | 0.2% |
| ichthyosis | 1 | 0.2% |
| keloid | 1 | 0.2% |
| leukoplakia | 1 | 0.2% |
| lichen planus | 1 | 0.2% |
| malassezia furfur | 1 | 0.2% |
| melasma | 1 | 0.2% |
| neurofibromatosis | 1 | 0.2% |
| onychocryptosis | 1 | 0.2% |
| skin cancer (unspecified) | 1 | 0.2% |
| skin symptoms of sclerosis multiplex | 1 | 0.2% |
| systemic lupus erythematosus | 1 | 0.2% |
| striae | 1 | 0.2% |
| unknown*** | 4 | 0.6% |

*Patients were allowed to report multiple skin conditions.
** Other than onychomycosis and tinea pedis.
***Patients that described their skin symptoms, but did not report the name of the condition.
